# Reproducibility in systems biology modelling

**DOI:** 10.15252/msb.20209982

**Published:** 2021-02-23

**Authors:** Krishna Tiwari, Sarubini Kananathan, Matthew G Roberts, Johannes P Meyer, Mohammad Umer Sharif Shohan, Ashley Xavier, Matthieu Maire, Ahmad Zyoud, Jinghao Men, Szeyi Ng, Tung V N Nguyen, Mihai Glont, Henning Hermjakob, Rahuman S Malik‐Sheriff

**Affiliations:** ^1^ European Molecular Biology Laboratory European Bioinformatics Institute (EMBL‐EBI) Wellcome Genome Campus Hinxton, Cambridge UK; ^2^ Babraham Institute Babraham Research Campus Cambridge UK; ^3^ Beijing Institute of Lifeomics National Center for Protein Sciences (The Phoenix Center) Beijing China

## Abstract

Reproducibility of scientific results is a key element of science and credibility. The lack of reproducibility across many scientific fields has emerged as an important concern. In this piece, we assess mathematical model reproducibility and propose a scorecard for improving reproducibility in this field.

## Graphical abstract

Reproducibility of scientific results is a key element of science and credibility. This piece assesses the reproducibility of mathematical models and presents an 8‐point scorecard for improving model reproducibility.

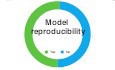

A survey of 1,576 scientists published in Nature (Baker, 2016) reported that over 70% of the participants failed to reproduce others' experiments and over 50% failed to reproduce their own results. It was assumed that systems biology modelling would remain relatively untouched by the reproducibility crisis, as the models are a specific set of computational codes representing well‐defined mathematical equations to perform reproducible simulations. However, mathematical models from a number of published articles were shown not to reproduce the simulation results described in the article (Mendes, 2018). To assess model reproducibility and identify the major causes of failure, we systematically analysed 455 mathematical models in conjunction with the curation process in the BioModels repository. Remarkably, about half of the published models were not reproducible either due to incorrect or missing information in the manuscript. We propose an 8‐point reproducibility scorecard for modellers, reviewers and journal editors to assess models and address the reproducibility crisis.

Experimental results fail reproducibility tests due to several reasons including improper documentation of methodology, considering noise as a positive finding, unrecognized or incomplete experimental variables, data fabrication or bias and publishing premature or incomplete results (Baker, 2016; Munafò *et al*, 2017). Computational biology research also faces reproducibility issues, compounded by several factors including changes in reference data and/or formats, software versions and missing essential codes or methodology (Schnell, 2018; Papin *et al*, 2020). Several suggestions have been published to improve reproducibility in bioinformatics (Kim *et al*, 2018). Systems biology modelling involves mathematical representation of biological processes to investigate complex system behaviours, which cannot be studied by looking at individual components (Le Novère, 2015). While lack of reproducibility of experimental results and computational analyses could be due to several compounded factors, lack of reproducibility in the simulation of mathematical equations in systems biology models is typically the result of inadvertent error or lack of information in the manuscript. In order to address the reproducibility crisis, it is therefore critical to pinpoint the precise causes of models’ failure. Moreover, it is important to assess how prevalent the lack of reproducibility is in systems biology modelling.

BioModels (https://www.ebi.ac.uk/biomodels/) is one of the largest public open‐source databases of quantitative mathematical models, where the models are manually curated and semantically enriched. Here, we present a systematic analysis of model reproducibility, coordinated with curation in BioModels, by attempting to independently reproduce published modelling results. In total, we investigated 455 published ordinary differential equation (ODE) models of various biological processes. These models were not randomly sampled but selected based on BioModels’ curation priorities driven by funding, collaborations, curators’ interest and direct submissions to BioModels. While not randomized, our sample covered models from a wide range of life science fields taken from articles published in 152 journals. The 455 models represent over 20% of all public literature‐based models available in the BioModels database.

## Model reproducibility assessment

The manual curation of models in BioModels involves a two‐step process: encoding models in standard formats and reproducing the simulation figures in the reference manuscript followed by semantic enrichment of the model and its components (Malik‐Sheriff *et al*, 2020). Semantic enrichment of the model in BioModels was done following the MIRIAM guidelines and it involved annotation of model entities (species, reactions, parameters, events, etc.) with cross‐references to controlled vocabularies such as GO (Gene Ontology), ChEBI, Mathematical Modelling Ontology, Systems Biology Ontology, Brenda Tissue Ontology and Experimental Factor Ontology, as well as data resources such as UniProt, Ensemble, NCBI Taxonomy and Reactome.

The following steps were employed to assess the reproducibility:

1. The manuscript was carefully read, and the model equations were encoded in the standard SBML format. When the models were previously submitted in SBML format, the equations, values of parameters and initial concentration, perturbation events, etc. were cross‐verified with the reference manuscript. The simulations of SBML model files were performed predominantly using COPASI (http://copasi.org/).
2. When COPASI was used in the original manuscript, other simulation software such as SimBiology toolbox (MATLAB) (https://www.mathworks.com/), libSBMLsim (https://fun.bio.keio.ac.jp/software/libsbmlsim/) and Mathematica (https://www.wolfram.com/mathematica/) were used to perform simulations.
3. The model was considered as reproducible when it reproduced at least one of the main simulation figures in the associated research article using a software different from the one used in the original manuscript. The reproduced simulation figure, such as time‐course plot with and without perturbation and phase‐plane plot, should match the original figure, and any minor deviation was still considered acceptable if it did not affect the scientific conclusion of the study. The models which could be directly reproduced with the description in the manuscript were labelled as “Directly Reproducible”.
4. When the model failed to reproduce the simulation with the mathematical equation and the parameter values provided in the research article, we resorted to an empirical trial and error approach to correct the model based on curator expertise. For example, any terms missing in the equations but described in the manuscript were added to correct the model; any potential typos such as misplacement of decimal points in the parameter values were corrected. The models that were reproduced after such corrections were labelled “Reproduced with manual corrections”.
5. The authors of the failed, yet potentially salvageable models were contacted when possible and their responses were recorded. The models that were reproducible with the corrections provided by them were labelled as “Reproduced with author support”.
6. Models that still could not be reproduced were labelled as “Non‐reproducible” and the likely reasons were recorded. The plausible reasons for non‐reproducibility include (i) inconsistency in model structure, i.e. any error in the model equation, (ii) missing parameter values, (iii) missing initial concentration and (iv) unknown reason.
7. SBML representations of all the analysed models were submitted to BioModels (Malik‐Sheriff *et al*, 2020). The reproducible ones were labelled as curated models.

## 49% of the published models were not directly reproducible

**Figure 1:**
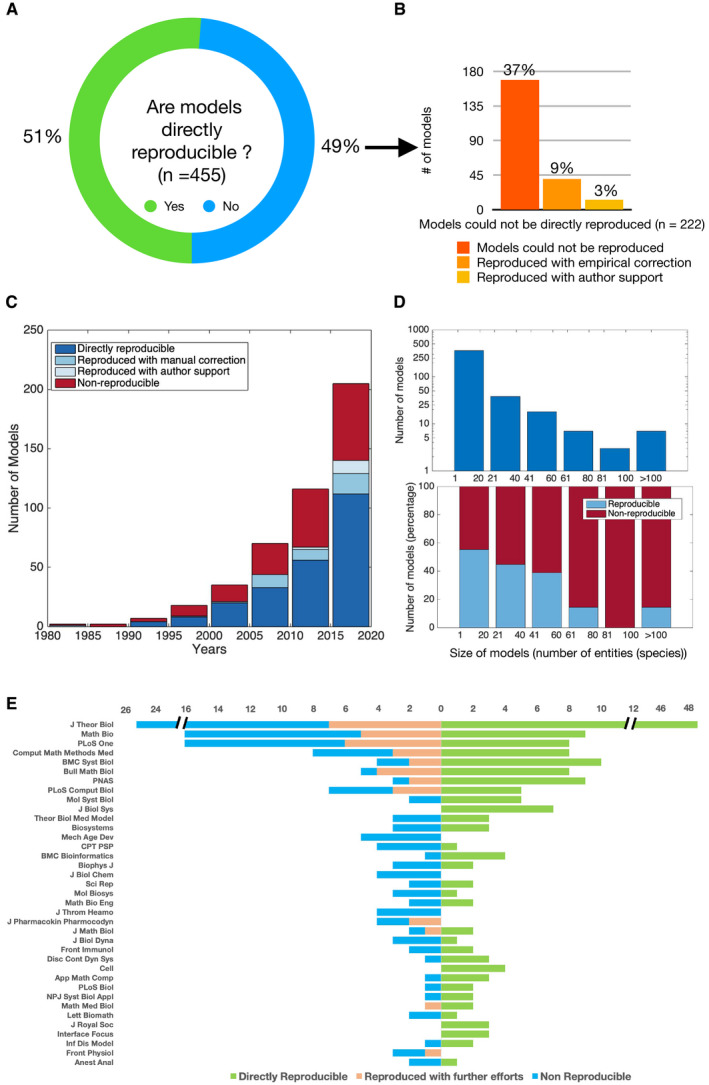
Reproducibility of systems biology models (A) About half of the published systems biology models could not be directly reproduced. (B) About 12% of the models could be reproduced with empirical corrections or author support. (C) The year of publication of the models analysed in this study. (D) Distribution of model size (number of entities/species) (top) and percentage of reproducible (directly reproducible and reproduced with further efforts) and non‐reproducible models (bottom) at specified bins of model size. (E) Distribution of directly reproducible, reproduced with further efforts (empirical correction and author support) and non‐reproducible models across various journals with more than two models in our work.

In total, we analysed 455 kinetic models among which the mathematical equations of 389 models were manually encoded in the standard SBML format using COPASI from the original manuscript. The remaining 66 models were those submitted to BioModels in SBML format by the authors and hence they were carefully cross‐checked to ensure whether the mathematical equations, initial conditions and parameters were accurately represented. There are no limitations from SBML to encode and simulate the simple ODE models selected in this study. The SBML representation of 233 out of 455 models (51%) directly reproduced the simulation results in the original manuscript (Fig 1A). About 49% of the published models were not reproducible either due to incorrect or missing information in the manuscript. This high proportion was unexpected and exposed a serious issue within the field. Even among the 66 models submitted to BioModels in SBML standard format, only 37 could be reproduced directly. The full list of 455 models, the link to respective SBML code in BioModels and their reproducibility status, are provided at https://www.ebi.ac.uk/biomodels/reproducibility.

## 12% of the models could be reproduced with further effort

About 12% of all models could be reproduced with further efforts involving either a careful empirical trial and error approach or author support (Fig 1B). Forty models (9%) were successfully reproduced with manual empirical correction of the inaccurate reporting in the manuscript. Some of the common errors that could be identified and manually corrected were (i) error in the sign of the terms in the mathematical equations, e.g. a negative sign for a production term in the equation or vice versa; (ii) missing terms in the model equations—e.g. missing one of the production or depletion terms in the ODE definition; (iii) typos in the parameter values, e.g. mistakes in the decimal points, a value of 0.01 was reported in place of 0.001; (iv) missing values, e.g. some missing initial concentrations of model entities could be inferred from the initial time point in the simulation plots; and (v) error in the units of initial concentration and parameter values, e.g. nmol/l was misrepresented as µmol/l.

It was not always possible to estimate the missing concentration, parameter or mathematical expression. In these cases, we contacted the corresponding authors to request the missing information or seek clarification. This was not always feasible, for example, due to authors’ change of institutions, change of field, leaving academia and death. In total, we attempted to contact the corresponding authors for 90 models, among which less than a third (27) responded. About half of the models of authors who responded (13 models, or 3% of the total number of models) were subsequently reproduced. These were mostly models published in the last 5 years (Fig 1C). Surprisingly about 70% of the authors we contacted did not respond to the request to provide information to reproduce their models. Our analysis included models with a wide range of sizes (3–200 entities) (Fig 1D), published in major life science journals (Fig 1E). About 63% of the models could ultimately be reproduced, combining those reproduced directly with the information provided in the manuscript and those with further efforts.

## The major reasons why models failed to reproduce

About 37% of the 455 models (*n* = 169) could not be rescued even after further efforts. The main reason why some of these models (*n* = 99) failed to reproduce was missing parameters values (*n* = 52), followed by missing initial conditions (*n* = 44) and inconsistency in model structure (*n* = 36), or a combination of the aforementioned causes. Among these 99 models, two failed due to all the three reasons; 19 due to missing parameters and initial conditions; six due to missing parameters and inconsistent model structure; and four due to missing initial conditions and inconsistent structure. Yet, in a large proportion (*n* = 70) of the non‐reproducible models, the reason for failure was unclear. The reference manuscripts of those models might have reported incorrect parameter values, initial concentrations or model equations or a combination of these three factors. Some research articles report parameter values in the form of plots, and it was not straightforward to extract those values. Insufficient and incorrect reporting of the model content were the main reasons why models failed to reproduce. These factors are commonly overlooked in the peer‐review process and hence the lack of reproducibility is reflected across journals from several life science fields (Fig 1E).

## Take‐home messages and recommendations

Overall, our analysis indicated that about half of the examined models cannot be reproduced using the information provided in the manuscript. Although the lack of reproducibility has been discussed within the community (Mendes, 2018; Papin *et al*, 2020), it was not expected to be this adverse. Given that the inability to reproduce models is widespread in research articles published across several journals with an exception of few (Fig 1E), it is imperative to revisit the peer‐review process of mathematical studies as previously suggested (Schnell, 2018).

Reproducibility, replicability and repeatability are terminologies often confused and defined differently in experimental and computational research (Miłkowski *et al*, 2018). In the context of systems biology modelling, the refined definition of replicability (also referred as repeatability) is the ability to use the same code provided with the manuscript in the same software to reproduce the simulation results, whereas reproducibility is the ability to build the code *de novo* and/or ensure the mathematical expressions are correctly represented and reproduce the simulation results in a software different from the one originally used. The focus of this work was to assess the reproducibility of the published systems biology models and hence the latter was chosen as the criterion.

Even in the models that are reproduced, one of the challenges we faced is unambiguous inference of the model entities and their values. When a variable or model entity name is different in the main manuscript description, mathematical expression and code, it becomes challenging to match them to reproduce the simulation. For example, “alpha” in the model description and/or equation in the manuscript and the code may refer to completely different entities. We managed to overcome this challenge by carefully reading the reference manuscript. We strongly recommend making the code or the model file as self‐contained as possible with proper annotation of the model entities. Model codes written in programming languages such as MATLAB, python, C and R are often helpful to reproduce the model. Nevertheless, not all such codes are easily comprehensible, especially when they are not well commented. Although the systems modelling community is split, a notable fraction of the modellers use COMBINE community standard formats such as SBML, SED‐ML and COMBINE Archive to encode their models. These standard formats provide a consistent framework to encode and annotate models, making them both human and machine‐readable. The strong community support for standard formats such as SBML makes it highly interoperable with about 280 supporting software tools for model construction, simulation, visualization and processing the semantic layer. We highly recommend using standard formats to encode and disseminate mathematical models as these greatly enhance the ability to comprehend and reproduce the models.

The most common approaches in systems biology modelling include kinetic, constraint‐based, logic and agent‐based modelling. Here, we specifically focused on ODE models, one type of kinetic models, and observed that half of these relatively simple deterministic kinetic models could not be reproduced. Other types of kinetic models include delay, partial and stochastic differential equations; they are likely to be affected either to the same extent or even more by the lack of reproducibility as they are somewhat more complex compared to ODE models. Moreover, the models that appeared to be non‐reproducible in a first curator assessment were less likely to be selected for curation in BioModels. For these reasons, our result suggesting the lack of reproducibility in 49% models is more likely to be an underestimate. We also compared the size of the model (i.e. number of entities in a model) with its reproducibility, and Mann–Whitney U‐test using MATLAB function rank sum showed that the distribution of the size of reproducible models is significantly different from the size of the non‐reproducible models with *P*‐value < 0.0001. Obviously, the relatively smaller models are slightly more reproducible than the larger ones. However, a significant proportion of the small models could not be directly reproduced (Fig 1D).

In the case of constraint‐based modelling, flux values resulting from flux balance analysis are commonly reported in manuscripts and these values are not unique solutions and cannot be directly reproduced. The community tool MEMOTE was developed primarily to quality control constraint‐based models. We are currently collaborating with constraint‐based modellers to develop tools and procedures to test the reproducibility of these models. Similarly, we have engaged with the logic modelling community to develop guidelines for curation and annotation of logic models (CALM).

Model curation is a time‐intensive task. On average, it took about a week to carefully encode and thoroughly investigate the reproducibility of a single model; in some cases, it took less than 2 days and in some cases over 2 weeks. Our criterion for a reproducible model was that it should reproduce at least one figure from the original article, which we consider to be a reasonable compromise between reliably assessing reproducibility, and the huge additional effort that would be required to ensure reproducibility of all relevant figures. One potential reason for a model to be classified as non‐reproducible is curator error. However, our curators work as a team, and will consult with each other in particular in case of non‐reproducibility. Moreover, if a curator, who works full time on curating models from the scientific literature, cannot reproduce a model, then a scientist who only occasionally tries to use a model from the literature is in our opinion unlikely to fare better than the curator. Further, among the 66 models directly submitted to BioModels in SBML by the authors, the rate of non‐reproducibility was similar, and 29 models (44%) could not be directly reproduced. Thus, we believe that the rate of misclassification due to curator error, while not zero, is highly unlikely to alter the conclusions of our analysis.

Our analysis was not intended to call‐out non‐reproducible models, but rather to assess the current status and the reasons behind the lack of reproducibility. Thereby, we intend to raise community awareness among the researchers who use systems biology models and make a potential contribution towards addressing the reproducibility crisis. We provide the “curated” section of BioModels as a source of reliable, verified reproducible models, while still keeping the non‐reproducible models accessible. The versioning system in BioModels will allow authors to improve their models, while keeping the original version accessible as a part of the public record. Currently, we keep the non‐reproducible models in the “non‐curated” part of BioModels, which also contains models from direct submissions which are still awaiting curation, as well as models in representations for which we currently do not provide detailed curation. We do not explicitly label non‐reproducible models because, on the one hand, there is a chance that the failure to reproduce the model is due to curator error and, on the other hand, we do not want to discourage authors from making their models accessible through a public repository. However, we are aware that the lack of explicit labelling of a non‐reproducible model might cause others to try the same again. During an open discussion in a dedicated breakout session on this work at COMBINE 2020 (http://co.mbine.org/), the modelling community recommended a transparent and flexible labelling of non‐reproducible models in BioModels.

## Reproducibility scorecard

Leveraging on the lessons learned from attempting to reproduce 455 models as well as our interaction with modelling communities, we have developed a simple reproducibility scorecard to enhance the ability to reproduce systems biology models (Box 1). The scorecard consists of a list of items that would help another modeller to reproduce the simulation results of a model with a reasonable effort. We recommend authors, reviewers and journal editors to assess each systems biology model in a research article using this scorecard. The scorecard consists of eight questions with a unit score for each “yes” as an answer. All eight questions may not always be applicable and hence, on the scale of 8, we strongly advocate that a model get a score of 4 points at the least.

Box 1 **Reproducibility scorecard**

1. Are the mathematical expressions described in the manuscript/supplementary material?
2. Are the parameters and entity initial levels listed (as a table) in the manuscript/supplementary material?
3. Are simulation conditions including software/programming environment, algorithm, changes in parameters/concentration/states and any data normalization described under each simulation figure or attached as a supplementary material?
4. Are the model code(s) for the mathematical expression and simulation shared publicly?
5. Are the model codes available in standard formats such as SBML, COMBINE archive, SED‐ML and are syntactically validated?
6. Are the model codes deposited in a relevant open model database?
7. Are the model codes well documented to unambiguously identify model entities/variables? (with additional annotation of reactions, mathematical expressions, events, conditions, etc. when relevant.)
  Are the models in standard formats such as SBML and COMBINE Archive are semantically enriched, i.e. annotated with controlled vocabularies such as Gene Ontology and ChEBI and database resources such as Gene Ontologies?
8. Are the numerical results shared publicly along with the model codes?
  Total Score (out of 8)

Among the 37% non‐reproducible models, 22% (99 models) could not be reproduced due to the three main reasons: inconsistency in model structure, missing initial concentration and parameters values. In another 15% (70 models), the reason is unknown, suggesting that one or more of the aforementioned information is reported incorrectly. Hence, a clear and complete description of the mathematical model, relevant parameter values and simulation conditions is vital to reproduce the model and is addressed in the first three questions of the scorecard. The model description in the manuscript or supplementary material is often useful for troubleshooting and to rectify any discrepancy in the model code or vice versa when available. A model is often simulated under multiple scenarios and therefore information such as the type of simulation (e.g. time course, steady‐state simulation for kinetic models) and solver/software used; changes in parameters, initial levels of model entities or reactions should be clearly described for each simulation figure in the manuscript or supplementary material. The reason why we emphasize is that some manuscripts list model parameters and initial levels for the base model as a table but fail to provide the changes to these values for different simulation scenarios. In case of large models, it may not be possible to enumerate all the aforementioned information in the manuscript; hence, it seems acceptable to share the code publicly either via supplementary material, GitHub or a model repository, as covered in question 4. Unarguably, code availability will be helpful in reproducing the model. Following the Findable, Accessible, Interoperable and Reusable (FAIR) principles, we have recommended submission of the model code in standard formats and into the relevant model repositories in questions 5 and 6, respectively. However, question 4 is not redundant, as it is used to cover those modellers who use non‐standard formats, for example a collection of programming scripts, and share them via GitHub, unstructured repositories or similar. A model shared in standard format such as SBML and CellML gets an additional point in our scorecard, as the interoperability of the model file will provide the possibility to test and reproduce it using several supporting software tools.

Box 2 **Further Reading**

**Reproducibility in science**

Additional references to highlight the reproducibility crisis in different areas of science and suggestions to improve reproducibility.

Begley CG & Ioannidis JPA (2015) Reproducibility in Science. *Circ Res* 116: 116–126

Fanelli D (2018) Opinion: Is science really facing a reproducibility crisis, and do we need it to? *Proc Natl Acad Sci* 115: 2628–2631

Goodman SN, Fanelli D & Ioannidis JPA (2016) What does research reproducibility mean? *Sci Transl Med* 8: 341ps12‐341ps12

Pusztai L, Hatzis C & Andre F (2013) Reproducibility of research and preclinical validation: problems and solutions. *Nat Rev Clin Oncol* 10: 720–724

Stodden V, Seiler J & Ma Z (2018) An empirical analysis of journal policy effectiveness for computational reproducibility. *Proc Natl Acad Sci* 115: 2584–2589

Sandve GK, Nekrutenko A, Taylor J & Hovig E (2013) Ten Simple Rules for Reproducible Computational Research. *PLOS Comput Biol* 9: e1003285

Schaduangrat N, Lampa S, Simeon S, Gleeson MP, Spjuth O & Nantasenamat C (2020) Towards reproducible computational drug discovery. *J Cheminformatics* 12: 9

**Modelling guidelines/community efforts**

**MIRIAM** (Minimum Information Requested in the Annotation of Biochemical Models)

Le Novère N, Finney A, Hucka M, Bhalla US, Campagne F, Collado‐Vides J, Crampin EJ, Halstead M, Klipp E, Mendes P, *et al* (2005) Minimum information requested in the annotation of biochemical models (MIRIAM). *Nat Biotechnol* 23: 1509–1515

**CALM** (Curation and annotation of Logical models).

Niarakis A, Kuiper M, Ostaszewski M, Malik Sheriff RS, Casals‐Casas C, Thieffry D, Freeman TC, Thomas P, Touré V, Noël V, *et al* (2020) Setting the basis of best practices and standards for curation and annotation of logical models in biology—highlights of the [BC]2 2019 CoLoMoTo/SysMod Workshop. *Brief Bioinform* https://doi.org/10.1093/bib/bbaa046

**MEMOTE** (Metabolic Model Testing)

Lieven C, Beber ME, Olivier BG, Bergmann FT, Ataman M, Babaei P, Bartell JA, Blank LM, Chauhan S, Correia K, *et al* (2020) MEMOTE for standardized genome‐scale metabolic model testing. *Nat Biotechnol* 38: 272–276

**COMBINE** (COmputational Modeling in BIology Network)

Waltemath D, Golebiewski M, Blinov ML, Gleeson P, Hermjakob H, Hucka M, Inau ET, Keating SM, König M, Krebs O, *et al* (2020) The first 10 years of the international coordination network for standards in systems and synthetic biology (COMBINE). *J Integr Bioinforma* 17: 20200005

**Reproducible Bioinformatics Project** (RBP)

Kulkarni N, Alessandrì L, Panero R, Arigoni M, Olivero M, Ferrero G, Cordero F, Beccuti M & Calogero RA (2018) Reproducible bioinformatics project: a community for reproducible bioinformatics analysis pipelines. *BMC Bioinformatics* 19: 349

Similarly, deposition of models to open repositories including, but not limited to, BioModels, Physiome (https://www.physiome.org) or JWSOnline (https://jjj.mib.ac.uk) will also get an additional score as they promote FAIR sharing. The advantage of submitting models to open model repositories includes provision of (i) a sophisticated search engine to make models findable, (ii) a version‐controlled storage system to make the models readily accessible, (iii) support for interoperable standard formats and (iv) curation and annotation services to promote reproducibility and thereby reusability. BioModels, being the largest repository of open, curated, well‐annotated and findable models, contributes significantly to reproducibility (Mendes, 2018). Several journals recommend authors to submit their model to BioModels. Similar to the curation service in BioModels, JWSOnline and the Physiome repository through the Centre for Reproducible BioMedical modelling (Papin *et al*, 2020) provide expert curation services to validate published reports. Therefore, a model code publicly shared in a non‐standard format in an unstructured repository will get one point, whereas a model code in standard format shared via open model repositories will get three points.

Unambiguous identification of the model entities is critical to reproduce the results; hence, semantic enrichment or proper documentation of the code as referred to in question 7 brings added value. Even an accurately defined model cannot be reproduced if the data normalization and the simulation conditions which include changes to specific parameters or concentration of model entity are not clearly described. We recommend providing this information under each related figure. Alternatively, the modellers can submit SED‐ML files, a COMBINE standard for description of simulation experiments. Although it is possible to use the simulation figures in the manuscript as a reference to test the reproducibility of the models in many cases, it is desirable to provide the numerical output of the simulations to verify the model reproducibility when appropriate, as covered in question 8.

**Figure 2:**
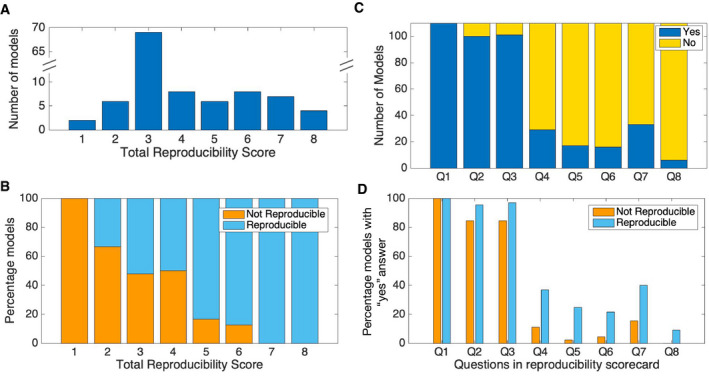
The 8‐point reproducibility scorecard is an indicator of model reproducibility (A) Distribution of total reproducibility score across models*. (B) Percentage of non‐reproducible and reproducible models for each total score. (C) Distribution of points scored by models* from each question in the scorecard. Each “yes” answer to a question in the scorecard will gain a score of 1. (D) Comparison of points scored by non‐reproducible and reproducible models from each question. *110 models (45 Not reproducible and 65 reproducible**) from the 455 models were scored using the scorecard. **Reproducible models include both directly reproducible models (*n* = 46) and those reproduced with further efforts (*n* = 19). Q1 – Q8 are questions representing 8 points in the reproducibility scorecard.

To demonstrate the applicability of the 8‐point reproducibility scorecard, we randomly selected 110 out of 455 models, scored their status prior to curation and assessed whether the total score and individual questions in the scorecard are associated with the model reproducibility (Fig 2). A majority of the models received a total score of 3 (Fig 2A). As the score increased, the percentage of reproducible models also increased (Fig 2B). A chi‐square test of independence showed a significant association between score ≥ 4 and model reproducibility, *X^2^* (1, *N* = 110) = 10.0733, *P* = 0.0015, odds ratio = 4.62 (95% CI: 1.71, 12.44). This result supported our recommendation of 4 point cut‐off in the reproducibility scorecard. Furthermore, a chi‐square test of independence showed that questions Q2 to Q8 are significantly associated with model reproducibility with *P* < 0.05. Although Q1 is highly relevant for reproducibility, *X^2^* could not be calculated because all the 110 models (including both reproducible and non‐reproducible) had “yes” as an answer for question 1 in the scorecard, meaning that curators in BioModels chose models with mathematical equations described in the manuscript or supplementary material (Fig 2C and D).

In parallel to the reproducibility scorecard, others have recently proposed recommendations for best practices (Porubsky *et al*, 2020) and specialized peer review of models (Papin *et al*, 2020). These approaches and our proposed scorecard are not mutually exclusive and can be employed individually or in combination to improve reproducibility in systems biology models, a key goal at the forefront of the current community discussion. By including our scorecard in the peer‐review process, complemented by the curation services provided by model repositories and reproducibility centres, we believe that the reproducibility crisis can be addressed. This crisis can be tackled as a community, where authors, reviewers and journal editors embrace reproducibility more proactively than before.

## Conflict of interest

The authors declare that they have no conflict of interest.
